# Evolution in the Surgical Management of Gastric Cancer Peritoneal Metastases

**DOI:** 10.3390/cancers17010100

**Published:** 2024-12-31

**Authors:** Matthew Krell, Suedeh Ranjbar, Saige Gitlin, Diego R. Alvarez Vega, Rachel Wilson, Kenya Thrasher, Zachary J. Brown

**Affiliations:** Department of Surgery, Division of Surgical Oncology, NYU Langone Health, NYU Grossman Long Island School of Medicine, Mineola, NY 11501, USA; matthew.krell@nyulangone.org (M.K.); saige.gitlin@nyulangone.org (S.G.); diego.alvarezvega@nyulangone.org (D.R.A.V.); rachel.wilson@nyulangone.org (R.W.); kenya.thrasher@nyulangone.org (K.T.)

**Keywords:** gastric cancer, peritoneal metastasis, cytoreductive surgery, HIPEC, PIPAC

## Abstract

Despite therapeutic treatments and the growing utilization of multimodal therapies, gastric cancer (GC) remains a highly aggressive malignancy with high mortality worldwide. Much of the complexity in treating GC is due to the high incidence of peritoneal metastasis (PM), with mean overall survival typically ranging from 4 to 10 months. With current systemic therapy, targeted therapies, and immunotherapies continuing to remain ineffective for GC/PM, there has been a significant growing interest in intraperitoneal (IP) therapies for the treatment of GC/PM. In this review, we summarize the development of PM and evolving treatment strategies for GC/PM. Furthermore, we explore the various advancements and outcomes of IP therapies, including heated intraperitoneal chemotherapy (HIPEC), neoadjuvant HIPEC, and pressurized intraperitoneal aerosolized chemotherapy (PIPAC).

## Simple Summary

Gastric cancer (GC) is a deadly disease with a high mortality rate, particularly when it spreads to the peritoneum, a condition known as peritoneal metastasis (PM). Current treatments are not very effective, and patients with GC/PM often have a poor prognosis with survival of only 4 to 10 months. This review explores emerging therapies for peritoneal metastases directly, including heated intraperitoneal chemotherapy (HIPEC) and newer methods like pressurized intraperitoneal aerosolized chemotherapy (PIPAC). The goal is to provide more effective treatment options for patients with GC/PM to improve survival rates and quality of life. The findings could lead to better treatment strategies and offer valuable insights to researchers in the field.

## 1. Introduction

Gastric cancer (GC) is a highly aggressive malignancy and is the fifth most frequently diagnosed cancer worldwide [1]. Due to high rates of diagnosis at an advanced stage, mortality rates remain high, making GC the third most common cause of cancer-related death globally [2]. The only potentially curative treatment approach for patients with GC is complete surgical extirpation [1,3]. Multimodal therapy with a combination of chemotherapy and surgery, with or without radiation therapy, has demonstrated improved outcomes for patients with GC [4,5]. However, many patients with GC present with or develop metastatic disease, which leads to high mortality [6]. In particular, the high incidence of peritoneal metastasis (PM) leads to markedly poor outcomes with a median overall survival (OS) of only 4–10 months [7,8]. The incidence of synchronous PM in patients with GC can be as high as 30%, and around 15–50% of patients may develop PM at the time of recurrence [7,8,9].

Despite advances in systemic chemotherapy, the OS for patients with GC/PM remains poor [10,11]. As a result, there has been great interest in intraperitoneal (IP) therapies for the treatment of GC/PM. IP treatment strategies include heated intraperitoneal chemotherapy (HIPEC), normothermic intraperitoneal chemotherapy (NIPEC) approaches, including early postoperative intraperitoneal chemotherapy (EPIC), and the emerging therapy of pressurized intraperitoneal aerosolized chemotherapy (PIPAC). In this review, utilizing a thorough search of PubMed, Embase, and clinicaltrials.gov, we discuss treatment strategies and advances in IP therapies for GC/PM.

## 2. Diagnostic Laparoscopy and Cytology Positive Disease

Peritoneal metastases are common in patients with GC, even if not appreciated on initial staging imaging [9,12]. The prognosis of patients with positive peritoneal cytology is dismal, and patients often do as poorly as those patients with gross PMs [13]. Diagnostic laparoscopy (DL) is a vital tool for detecting radiographically occult disease in patients with GC at high risk for developing PMs to accurately stage patients and avoid surgery that yields no survival benefit [12,14]. In a retrospective review from a single center, Ikoma et al. identified 711 patients who underwent laparoscopy, and 148 patients (20.8%) were found to have macroscopic PM. However, of the 514 patients without gross macroscopic PM, 68 patients (13.2%) had cytology-positive disease. Notably, patients with cytology-positive disease demonstrated poor outcomes with a disease-specific survival (DSS) of 1.3 years compared to a DSS of 0.8 years for patients with macroscopic PM [12].

Although there are known benefits of DL, it has been found to be underutilized. Using the Surveillance, Epidemiology, and End Results (SEER) population-based cancer registry, Karanicolas et al. found that of the patients who had any operation for GC, only 7.9% underwent a DL. Additionally, 29.8% of those patients who had a DL did not undergo a therapeutic operation [15]. Similarly, an updated analysis of SEER-Medicare-linked data from 2004 to 2013 found the use of DL remained low at 13% but increased annually from 6.4% to 22.2%. The performance of DL was associated with patient demographics, tumor location, and treatment at a National Cancer Institute (NCI) Designated Cancer Center [16].

The role of gastrectomy with lymphadenectomy in patients with GC-positive peritoneal cytology has been investigated and found to offer no survival benefit [17]. However, patients whose cytology converts from positive to negative during system therapy have improved survival. In a retrospective cohort study, Valletti et al. investigated patients at a single center between 2011 and 2019, classifying patients into four risk groups: no peritoneal disease, cytology positive who converted to negative cytology after systemic chemotherapy, cytology positive without conversion after chemotherapy, and patients with visible PM. In patients without peritoneal disease and in patients who converted from positive to negative cytology, the median OS was not reached, with a 3-year survival of 65% and 53%, respectively. In patients who did not convert from positive to negative cytology, the median OS was 13 months and was found to be 16 months in patients with gross PM, with no difference in OS between these groups (*p* = 0.364) [18]. Therefore, patients who demonstrate favorable disease biology may be candidates for surgical resection along with IP therapy.

## 3. Therapeutic HIPEC

While multimodal therapy and surgical extirpation offer curative potential in patients with early-stage GC, patients with PM are often treated with palliative intent [19]. Chemotherapy alone is used for palliative treatment of these patients and aims to prolong survival, delay progression, and improve symptoms, though patients rarely survive over one year on traditional chemotherapeutic regimens [20]. The role of gastrectomy without metastasectomy in patients with a single non-curable risk factor, such as metastatic disease to the liver, peritoneum, or para-aortic lymph nodes, was evaluated in the REGATTA trial. There was no difference in two-year OS between patients who received chemotherapy alone and those who underwent gastrectomy and systemic chemotherapy (31.7% vs. 25.1%, respectively). The median OS was 16.6 months in the chemotherapy alone group and 14.3 months in patients who received a gastrectomy (*p* = 0.70) [21]. As a result of the Regatta trial, routine gastrectomy without metastasectomy does not improve survival and is not performed for therapeutic indications.

**Figure 1:**
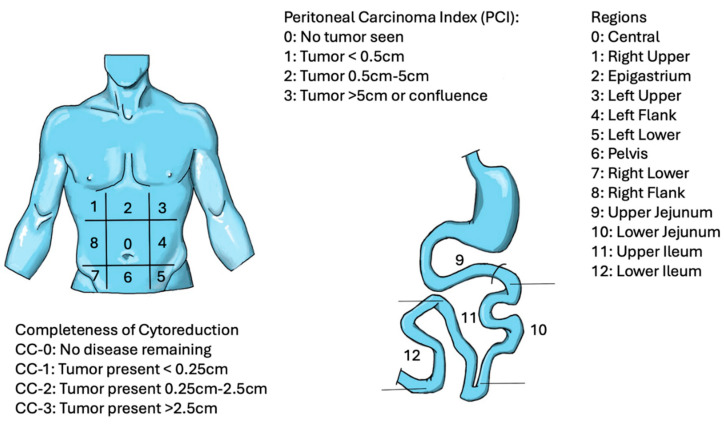
Peritoneal Carcinoma Index (PCI). The Peritoneal Carcinoma abdomen is calculated by dividing the abdomen into 13 regions (0–12). The PCI is measured by assigning a score to each region based on tumor size and taking the sum of all regions; higher numbers correspond with higher peritoneal disease burden. In addition to assessing the burden of disease, several scoring systems have been developed to assess the completeness of cytoreduction. As HIPEC has been demonstrated to be effective with residual disease less than 2–3 mm, complete cytoreduction has been defined as CC-0 or CC-1 resection with no residual disease or limited deposits <2.5 mm remaining, respectively.

Cytoreductive surgery (CRS) was first conceptualized nearly a century ago for the debulking of ovarian cancers [22,23,24]. Thereafter, a hyperthermic peritoneal perfusion system to deliver IP therapy was designed to deliver chemotherapy directly to the tumor surface [25]. In order to adequately select patients and to communicate effectively the extent of disease, Jacquet and Sugarbaker established the peritoneal carcinoma index (PCI) scoring system assessing tumor distribution and extent of disease within the abdomen [26,27,28] (Figure 1).

Recent years have called into question the utility of HIPEC across disease histologies [29]. The recent PRODIGE7 trial involving patients with PM from colorectal or appendix cancer demonstrated no difference in OS between CRS alone or CRS/HIPEC [29]. However, in patients with GC/PM, several studies have demonstrated the added benefit of HIPEC to CRS alone (Table 1). Yang et al. performed a phase III randomized controlled trial where patients with GC/PM were randomized to CRS alone or CRS/HIPEC. There was a significant increase in median survival in the CRS/HIPEC cohort compared to the CRS-alone group. On multivariable analysis, CRS/HIPEC, synchronous PM, complete cytoreduction, systemic chemotherapy ≥ 6 cycles, and no serious adverse events were independent predictors for improved survival [30]. Similarly, the more recent CYTO-CHIP study investigated CRS/HIPEC compared to CRS alone. From prospective databases, 277 patients with GC/PM were included: 180 underwent CRS/HIPEC and 97 CRS alone. Compared to CRS alone, CRS/HIPEC demonstrated improved median OS (18.8 versus 12.1 months) [31].

Compared to the previous studies that evaluated CRS vs. CRS/HIPEC, Rudloff et al. performed a prospective randomized control trial to compare systemic therapy alone versus CRS/HIPEC/systemic therapy. Patients were randomized to gastrectomy, metastasectomy, HIPEC, and systemic FOLFOXIRI versus FOLFOXIRI alone. Although closed for low accrual, 7 of 9 patients in the multimodality arm achieved a CC0 cytoreduction. The median OS was 11.3 months in the multimodality arm compared to 4.3 months in patients who received systemic therapy alone. Additionally, all patients who underwent surgery that survived greater than 12 months had an initial PCI less than or equal to 15 [32].

A consistent finding across studies in the treatment of GC/PM is the importance of complete cytoreduction and proper patient selection based on the burden of disease (PCI). Glehen et al. performed a retrospective multicenter study between 1989 and 2007 to evaluate the toxicity and prognostic factors in patients who had CRS and IP chemotherapy as either HIPEC and/or EPIC. With a median OS of merely 9.2 months, the only independent prognostic indicator by multivariate analysis was the completeness of cytoreductive surgery [33]. The GECOP study was a retrospective database evaluation of patients with GC/PM from 2006 to 2017. Eighty-eight patients with GC/PM were treated with CRS/HIPEC. The median PCI was 6, complete cytoreduction was achieved in 80 patients (90.9%), and the median OS was 21.2 months. On multivariate analysis, the extent of peritoneal disease was identified as the only independent factor that influenced OS. Patients who had a PCI of 6 or lower had a significantly longer median OS of 26.1 months compared to 18.9 months in patients with a PCI higher than 6 [34]. Similarly, the DVAG-HIPEC study was a retrospective analysis of the national German HIPEC registry. Two hundred thirty-five patients who had GC/PM were included with a median PCI of 8 (range 1–30). A complete cytoreduction was achieved in 121 patients (71.6%). The median OS for the cohort was 13 months, with a 5-year survival rate of 6%. Notably, the median OS differed according to the PCI. Patients with PCI from 0–6 (n = 74), 7–15 (n = 70), and 16–39 (n = 24) had a median OS of 18 months vs. 12 months vs. 5 months (*p* = 0.002) [35].

The PERISCOPE I study was a non-randomized phase I–II study to assess the safety and feasibility of HIPEC following systemic chemotherapy in patients with GC and limited PM. Patients underwent HIPEC with 460 mg/m^2^ hyperthermic oxaliplatin for 30 min, followed by normothermic docetaxel for 90 min with escalating doses [36]. The PERISCOPE II study is an ongoing randomized phase III study investigating gastrectomy with CRS/HIPEC compared to standard-of-care systemic chemotherapy. Patients are to have resectable cT3/cT4 GC and a PCI < 7 or positive peritoneal cytology. Patients will undergo HIPEC with 460 mL/m^2^ for 30 min, followed by docetaxel 50 mg/m^2^ for 90 min [37]. With HIPEC remaining promising due to its high complete cytoreduction rates and improved median OS, many HIPEC studies, including PERISCOPE II, are currently being conducted. (Table 2).

Studies have also assessed the role of HIPEC in a palliative setting for the treatment of malignant ascites. Facchiano et al. evaluated the role of laparoscopic HIPEC on five patients with gastric cancer and unresectable carcinomatosis. Regression of ascites and symptoms was observed in all five patients [38]. Similarly, Ba et al. performed laparoscopic HIPEC on 16 patients with malignant ascites from gastric cancer. Complete remission of ascites was seen in 14 patients and partial remission in 2 patients [39].

**Table 1:** Selected Prospective Intraperitoneal Chemotherapies Studies in Gastric Cancer.

Study	Setting	Intervention	Intraperitoneal Regimen	Outcome
Yang et al. [30]	Therapeutic	CRS alone vs. CRS/HIPEC	cisplatin 120 mg and MMC 30 mg for 60–90 min	median survival CRS/HIPEC 11.0 months vs. CRS alone 6.5 months (*p* = 0.046)
GYMSSA Rudloff et al. [32]	Therapeutic	systemic therapy versus CRS/HIPEC/systemic therapy	oxaliplatin 460 mg/m^2^	median OS 11.3 months in multimodality arm vs. 4.3 months systemic therapy alone
PERISCOPE I [36]	Therapeutic	phase I–II study	460 mg/m^2^ hyperthermic oxaliplatin and normothermic docetaxel	maximum tolerated dose 50 mg/m^2^ IP docetaxel.
Badgwell et al. [40]	Neoadjuvant	Phase II: laparoscopic HIPEC; patients whose PM resolved; gastrectomy was offered	MMC 30 mg and cisplatin 200 mg	median OS from the date of diagnosis of PM 30.2 months; median OS from first laparoscopic HIPEC 20.3 months
Badgwell et al. [41]	Neoadjuvant	Phase II: gastrectomy, CRS, and HIPEC for low-volume disease	MMC 30 mg and cisplatin 200 mg	median OS from the date of diagnosis of PM 24.2 months. Median OS from the date of CRS, gastrectomy, and HIPEC 16.1 months.
PIPOX01 [42]	PIPAC- palliative	Phase I/II	oxaliplatin	Median PFS was 6.1 months, and the median OS was 13 months
Struller et al. [43]	PIPAC- palliative	phase II	cisplatin 7.5 mg/m^2^ and doxorubicin 1.5 mg/m^2^ every 6 weeks	median OS was 6.1 months, and the median time to progression was 2.7 months
PIPAC-GA2 [44]	PIPAC- palliative	Phase II: safety and efficacy of bidirectional chemotherapy	Systemic chemotherapy initially for four cycles followed by PIPAC with 7.5 mg/m^2^ and doxorubicin 1.5 mg/m^2^	median survival 13 months; four complete and five partial pathological responses
PIPAC-OPC1 [45] PIPAC-OPC2	PIPAC- palliative	Data from GC patients included in the prospective PIPAC-OPC1 and PIPAC-OPC2	7.5 mg/m^2^ and doxorubicin 1.5 mg/m^2^	the median survival from PM diagnosis was 11.5 months and 4.7 months after the first PIPAC procedure

CRS: cytoreductive surgery; HIPEC: heated intraperitoneal chemotherapy; MMC: mitomycin C; OS: overall survival; IP: intraperitoneal; PM: peritoneal metastases; PIPAC: pressurized intraperitoneal aerosolized chemotherapy; PFS: progression-free survival.

**Table 2:** Selected actively accruing gastric cancer HIPEC clinical trials.

Study Title	Sponsor	Condition	Intervention
Hyperthermic Intraperitoneal Chemotherapy (HIPEC) in Locally Advanced Gastric Cancer	Wuhan University	Malignant Neoplasm of Stomach	Procedure: D2 radical gastrectomy Drug: SOX neoadjuvant or postoperative chemotherapy Procedure: Hyperthermic intraperitoneal chemotherapy (HIPEC)
Prophylactic Preoperative HIPEC in Advanced Gastric Cancer at High Risk of Peritoneal Recurrence	Jagiellonian University	Gastric Cancer	Combination Product: FLOT + HIPEC + Surgery Combination Product: FLOT + Surgery
Robotic Cytoreduction and Hyperthermic Intraperitoneal Chemotherapy for Treatment of Gastric Cancer With Limited Peritoneal Metastasis, ROBO-CHIP Study	Mayo Clinic	Laparoscopic Gastrectomy With D2 Lymphadenectomy Combined With Hyperthermic Intraperitoneal Chemotherapy (HIPEC) or Not	Procedure: Biospecimen Collection Drug: Cisplatin Procedure: Computed Tomography
Laparoscopic Gastrectomy With D2 Lymphadenectomy Combined With Hyperthermic Intraperitoneal Chemotherapy (HIPEC) or Not	The Affiliated Hospital of Qingdao University	Gastric Cancer	Procedure: Hyperthermic Intraperitoneal Chemotherapy (HIPEC)
Phase II Study of the Effects of Laparoscopic Hyperthermic Intraperitoneal Chemotherapy (HIPEC) in Patients With Advanced Gastric Cancer	University of Chicago	Gastric Cancer Peritoneal Carcinomatosis	Drug: Cisplatin Drug: Mitomycin
A Phase II Trial of Neoadjuvant Laparoscopic Hyperthermic Intraperitoneal Chemotherapy (HIPEC) With Chemoradiation	Icahn School of Medicine at Mount Sinai	Gastric Cancer Stomach Cancer	Drug: Paclitaxel Drug: Carboplatin Drug: Dexamethasone
HIPEC + FLOT vs. FLOT Alone in Patients With Gastric Cancer and GEJ (PREVENT)	Krankenhaus Nordwest	Gastric Cancer Gastroesophageal Junction Adenocarcinoma	Drug: 5-Fluorouracil Drug: Leucovorin Drug: Oxaliplatin
Comparative Study of Lobaplatin and Paclitaxel in Advanced Gastric Cancer Patients With D2 Surgery Combined With Hyperthermic Intraperitoneal Chemotherapy	Wuhan Union Hospital, China	Gastric Cancer Chemotherapy Effect Surgery	Drug: Paclitaxel Drug: Lobaplatin
PIPAC in Multimodal Therapy for Patients With Oligometastatic Peritoneal Gastric Cancer	Azienda Ospedaliera Universitaria Integrata Verona	Oligometastatic Gastric Adenocarcinoma	Combination Product: FOLFOX and PIPAC
Response Prediction of Hyperthermic Intraperitoneal Chemotherapy in Gastro-Intestinal Cancer	Technische Universität Dresden	Gastric Cancer Colon Cancer Peritoneal Carcinomatosis	Other: Establishment of organoid cultures and in vitro sensitivity testing

## 4. Neoadjuvant HIPEC

Survival outcomes for patients with GC/PM undergoing CRS/HIPEC are largely dependent on PCI and completeness of cytoreduction, indicating the importance of patient selection and disease biology. As survival outcomes remained poor, it appeared as though a one-time administration of IP chemotherapy was not sufficient. Badgwell et al. performed two phase II studies investigating the outcomes of HIPEC with or without CRS in patients with GC/PM (Table 1). In the initial study, patients with gastric or gastroesophageal junction adenocarcinoma and positive cytology or radiologically occult PM after systemic chemotherapy underwent laparoscopic HIPEC. In patients whose PM resolved, including conversion to negative cytology, gastrectomy was offered. Nineteen patients were enrolled, of which 6 had positive peritoneal cytology and 13 with macroscopic PM. Thirty-eight laparoscopic HIPEC procedures were performed on 19 patients, with 53% of patients receiving one HIPEC procedure. Seven patients had negative peritoneal cytology and no carcinomatosis. Five patients went on to receive a gastrectomy. The median OS from the date of diagnosis of PM was 30.2 months, and the median OS from the first laparoscopic HIPEC was 20.3 months [40].

The subsequent study examined the outcomes of gastrectomy, CRS, and HIPEC for low-volume disease. Twenty patients were enrolled: six with positive cytology and fourteen with macroscopic PM. Patients received a median of eight cycles of systemic chemotherapy and had at least one laparoscopic HIPEC. The median PCI at the time of gastrectomy/CRS/HIPEC was 2 (range 0–13). The median OS from the date of diagnosis of PM was 24.2 months. The median OS from the date of cytoreduction, gastrectomy, and HIPEC was 16.1 months, with 1-, 2-, and 3-year OS rates from the diagnosis of PM being 90%, 50%, and 28%, respectively [41].

It has been hypothesized that repeat administration of IP chemotherapy can downstage patients, improving outcomes after CRS/HIPEC. Lee et al. performed a retrospective analysis of advanced GC/PM with a conversion group comprised of 34 patients who underwent laparoscopic HIPEC as neoadjuvant IP chemotherapy followed by CRS/HIPEC. Fifteen patients were included in the CRS/HIPEC group who underwent CRS/HIPEC alone. A total of 23 patients received systemic chemotherapy, and the palliative group had 23 patients who received conservative therapy or palliative gastrectomy. The conversion group had a significantly improved OS compared to the CRS/HIPEC, chemotherapy alone, and palliative groups (*p* < 0.001). Additionally, the conversion group had a significantly decreased PCI and ascites [46]. Similarly, Canbay et al. investigated gastrectomy and HIPEC following a response to laparoscopic HIPEC with bidirectional IP and systemic chemotherapy. A total of 34 of 53 (64.15%) patients underwent CRS with gastrectomy and HIPEC, and a complete cytoreduction was performed in 22 patients. The median survival was 18.9 ± 13.4 months [47]. Yu et al. investigated neoadjuvant laparoscopic HIPEC, bidirectional IP, and systemic induction chemotherapy followed by CRS versus patients who went directly to CRS/HIPEC. The neoadjuvant HIPEC group demonstrated greater tumor clearance in terms of post-CRS PCI (6 vs. 14, *p* = 0.002) compared to the CRS group. The median survival was 20.0 months with the neoadjuvant approach and 8.6 months in the CRS group (*p* = 0.031). Factors associated with increased survival were the neoadjuvant protocol, age, and post-CRS tumor clearance. Notably, the neoadjuvant strategy improved survival with high disease burden [48].

## 5. Prophylactic HIPEC

Generally speaking, a small tumor burden or microscopic disease is easier to treat than a large burden of disease. Therefore, there has been a growing interest across multiple disease pathologies for prophylactic HIPEC to treat micro-metastatic disease and subsequently decrease the rates of PMs. Allievi et al. performed a single-center retrospective study evaluating the use of CRS/HIPEC in patients at high risk for recurrence, which the authors labeled the “adjuvant group” or patients with GC PM or positive cytology (therapeutic group) [49]. Patients underwent neoadjuvant systemic therapy and surgical resection consisting of gastrectomy, D2 lymphadenectomy, and CRS. In the adjuvant HIPEC group, the OS at 1 year was 85.7%, and at 3 years, 71.4%; the DFS at 1 year was 71.4% and 64.3% at 3 years. In the therapeutic CRS and HIPEC group, the OS was 60.3% at 1 year and 35.1% at 3 years, and the DFS was 38% at 1 year and 32.6% at 3 years.

Brenkman et al. performed a meta-analysis of the use of prophylactic HIPEC in patients with GC, and OS was 32–35 months after prophylactic HIPEC compared to 22–28 months after surgery alone. The 5-year survival rates are 39–87% after prophylactic HIPEC and 17–61% after surgery alone. Peritoneal recurrence occurred in 7–27% in the HIPEC group, compared to 14–45% after surgery alone [50]. Overall, the use of prophylactic HIPEC remains very promising for decreasing rates of PM, as well as increasing OS while decreasing peritoneal recurrence.

## 6. PIPAC and Future Directions

Generally, there was a misconception of high mortality and morbidity related to CRS/HIPEC. Due to advances in surgical technique, postoperative care, and better patient selection, adverse outcomes have decreased over the past years. This may result in a number of potentially salvageable patients not being appropriately evaluated and intervened upon. When evaluating the Medicare Standard Analytic File from 2013 to 2017 with PM from colorectal or appendiceal cancer, Aquina et al. found that only 3.3% of patients underwent CRS/HIPEC, but more strikingly, only 6.4% of patients were referred to a peritoneal surface malignancy surgeon [51]. Therefore, significant disparities exist in treatment and access to care for patients with PM. Further studies are required to better understand the disparities in access to peritoneal malignancy surgeons in patients with gastric cancer and other emerging therapies.

**Figure 2:**
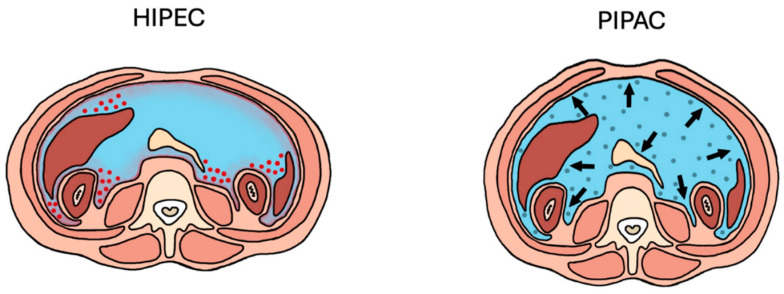
HIPEC and PIPAC. HIPEC involves the administration of chemotherapy in a heated perfusate to improve the efficacy of the chemotherapy. PIPAC delivers chemotherapy as an aerosolized substance under laparoscopic pressurization, leading to greater spatial distribution and tissue penetration of the drug.

Pressurized intraperitoneal aerosolized chemotherapy (PIPAC) has emerged as a safe and feasible treatment option for patients with PM [52]. PIPAC administers IP chemotherapy as an aerosol in combination with laparoscopy, thus delivering chemotherapy into the abdominal cavity in patients with unresectable or advanced PM [53]. There has been interest in utilizing PIPAC to treat patients with advanced PM, palliate symptoms, or induce regression of disease to convert to resectable disease [54]. PIPAC has been utilized in patients with gastrointestinal malignancies, including gastric cancer [42,55]. PIPAC also has the advantage that it can be safely administered laparoscopically for repeated drug delivery every 4–6 weeks in addition to systemic chemotherapy [56,57]. Furthermore, experimental evidence in animal models suggests that PIPAC as an aerosolized substance leads to greater spatial distribution and tissue penetration of the drug [58,59,60] (Figure 2).

PIPAC was first introduced as a method to treat PMs in late 2011. Since that time, multiple studies have evaluated the use of PIPAC to treat patients with GC PM (Table 1). The PIPOX01 study was a phase 1/2 study of PIPAC in patients who received at least three months of systemic chemotherapy and a PCI of more than 5 in gastric cancer, 13 in small bowel cancer, and 15 for colorectal cancer. Prior to enrollment, patients received a median of two chemotherapy lines and had a median PCI of 22.5. The median PFS was 6.1 months, and the median OS was 13 months [42].

Other prospective studies have investigated the utility of PIPAC in patients with GC/PM (Table 3). Struller et al. performed a phase II study of PIPAC in patients with GC PM after first-line palliative IV chemotherapy. PIPAC was found to be well tolerated, and 40% of patients experienced what the authors described as clinical benefit, including one patient who had a complete radiologic response and was alive 31 months after the first PIPAC. The median OS was 6.1 months, and the median time to progression was 2.7 months [43].

The phase II PIPAC-GA2 study assessed the safety and efficacy of bidirectional chemotherapy for GC/PM. PIPAC was performed every 6 weeks with XELOX administered between PIPAC treatments. A total of 31 patients were treated: 24 with synchronous PM at diagnosis and 7 with metachronous PM. The mean PCI was 13.8 (min–max 6–34), and 56 PIPAC procedures were performed. The median survival was 13 months, with four complete and five partial pathological responses seen in the 15 patients who were able to be evaluated [44]. Similarly, the PIPAC-OPC1 and PIPAC-OPC2 studies assessed cisplatin and doxorubicin PIPAC after systemic chemotherapy. The median PCI was 10.5. A total of 21 patients underwent 52 PIPAC procedures. With a median follow-up of 10.4 months, the median survival from the time of PM diagnosis was 11.5 months and 4.7 months after the first PIPAC procedure [45].

Retrospective studies have also evaluated the use of PIPEC in patients with GC/PM. Nadiradze and colleagues performed a retrospective analysis of 24 consecutive patients with GC/PM who underwent sixty PIPAC procedures. The median PCI was 16, and PIPAC was performed with cisplatin and doxorubicin at 6-week intervals. The median survival was 15.4 months. A total of 17 patients had repeat PIPAC procedures, with 12 patients having an objective tumor response [61]. Gockel and colleagues performed a retrospective review of a prospectively collected database between November 2015 and June 2018 on 24 patients with GC/PM. These 24 patients underwent 46 PIPAC procedures. PIPAC again consisted of combined cisplatin and doxorubicin. The median PCI prior to the first PIPAC was 14, and the median ascites volume was 100 mL. The median OS from the first PIPAC procedure was 121 days. Eight patients received more than three PIPAC procedures, with these patients having a median OS of 450 days. A total of 8 patients had a decrease or stable PCI at repeated PIPAC procedures, and of the 14 patients who underwent more than one PIPAC, 11 had decreased or stable ascites volume [62]. Similarly, Alyami and colleagues performed an analysis of their PIPAC database for patients with unresectable GC/PM before 2018. A total of 42 patients underwent 163 PIPAC procedures with a median PCI of 17. All patients had alternating systemic chemotherapy with cisplatin and doxorubicin PIPAC. The median consecutive PIPAC procedures were three. The overall survival was 19.1 months, and six patients (14.3%) became resectable during treatment [63].

In addition to finding tumor response with the potential to decrease PCI and ascites volume, PIPAC has also been found to positively impact patient quality of life. Li et al. performed a systematic review of the quality of life following PIPAC. Nine studies using the EORTC QLQ-C30 questionnaire to assess QoL after repeated PIPAC cycles were identified. Four studies were identified, including patients with PM GC or epithelial ovarian cancer (EOC). In 31 patients with GC and 104 EOC patients, QoL remained stable in 13/14 and 11/14 patients, according to the EORTC QLQ-C30 scales. PIPAC was inferior to CRS and HIPEC in global QoL and functioning but superior in symptom reduction [64]. Additionally, Odendahl et al. investigated QoL in 48 patients who underwent PIPAC every 6 weeks. After the first PIPAC, the global physical score deteriorated slightly after the first PIPAC but subsequently improved after the second PIPAC [65].

Although there has been progress in the treatment of GC, survival outcomes for patients with GC/PM remain poor. New innovative therapies and therapeutic combinations are required to improve patient outcomes. As of now, PIPAC is largely used in the palliative setting without additional surgical procedures being performed. The PLUS study is part of a retrospective review of international cohort studies evaluating the risks of additional surgical procedures performed at the time of PIPAC, including intestinal resections, gastrectomy, splenectomy, and bowel repair/stoma creation. Although there was a difference in the increase in surgical time (*p* < 0.001), length of stay (*p* < 0.001), and medical complication rate (*p* < 0.001), there was no difference in the rate of surgical complications [66]. The PIPAC EstoK 01 study is a prospective, randomized, multicenter phase II study evaluating doxorubicin and cisplatin PIPAC on patients with GC/PM with a PCI greater than 8. Patients are treated with two cycles of systemic chemotherapy between PIPAC procedures. The endpoints are 2-year OS, morbidity, quality of life, and secondary resectability rate [67].

In addition to improved therapies, a better understanding of the tumor pathophysiology may prove to improve clinical outcomes. The influence of the tumor microenvironment (TME) in patients with cancer is starting to be increasingly recognized. The TME not only consists of tumor cells but also associated stroma and immune cells. Recent preclinical studies investigated the role of the TME in patients with PMs in response to IP chemotherapy. Fujimori et al. investigated the TME in primary GC and GC/PM and reported that the number of CD8+ T cells was significantly less in PM lesions than in primary lesions (*p* < 0.01). Additionally, the number of immune-suppressive M2 macrophages was significantly increased in PMs (*p* = 0.016) [68]. Similarly, in patients with GC/PM, the proportion of CD8+ T cells, CD3+ T cells, NKT cells, and NK cells in the peritoneal fluid was significantly reduced in patients with PM compared to patients without PM [69]. Gou et al. investigated the antitumor activity of IP oxaliplatin in an abdominal implantation model of colon cancer, testing the effect on the local immune cell populations. IP administration of oxaliplatin increased tumor-infiltrating activated CD8+ T cells in tumors [70]. Similarly, Geva et al. performed IP inoculation of tumors and treated mice with MMC-based HIPEC, which increased overall survival (OS) compared to placebo-treated animals, which was accompanied by increased infiltration of CD8+, CD68+, and CD20+ cells. The addition of PD-1 treatment to HIPEC ameliorated survival compared to HIPEC alone (24.66 vs. 19.00 vs. 14.33 days, HIPEC + PD-1, HIPEC, and placebo, respectively; *p* = 0.008). This effect was accompanied by increased CD8+ T cell tumor infiltration [71]. As preclinical studies indicate, IP chemotherapy may increase the infiltration of immune cells to the TME and change the immune infiltrate into the TME, which may potentially translate into a therapeutic target. An improved understanding of tumor characteristics and intertumoral and metastatic heterogeneity may prove to improve patient outcomes with directed therapies targeting specific alterations. Additionally, combination therapies aimed at alterations induced by IP therapies may prove effective. More studies are required to further the field of IP therapies for PMs.

## 7. Conclusions

Peritoneal metastasis remains a formidable therapeutic challenge in gastric cancer, significantly contributing to its high mortality rates. Despite advancements in systemic chemotherapy and targeted therapies, the prognosis for patients with GC/PM remains poor. CRS combined with IP therapies, such as HIPEC, has shown promise in extending survival. Multiple studies have demonstrated that achieving complete cytoreduction and proper patient selection with a low PCI are critical factors linked to better prognoses. Additionally, neoadjuvant approaches have also shown potential in reducing tumor burden and converting unresectable disease to resectable. Large-scale, multicenter randomized controlled trials are crucial to validate these approaches and establish evidence-based guidelines. Ultimately, intraperitoneal therapies offer a valuable addition to the multimodal treatment strategy for G/PM, providing hope for improved survival and quality of life in this challenging patient population.

## Figures and Tables

**Table 3:** Selected actively accruing gastric cancer PIPAC clinical trials.

Study Title	Sponsor	Condition	Intervention
PIPAC and FOLFOX for Gastric Cancer Peritoneal Cancer	Vilnius University	Gastric Cancer Peritoneal Carcinomatosis	Drug: Combined Doxorubicin and Cisplatin Pressurized IntraPeritoneal Aerosol Chemotherapy With Systemic FOLFOX Chemotherapy
PIPAC in Multimodal Therapy for Patients With Oligometastatic Peritoneal Gastric Cancer	Azienda Ospedaliera Universitaria Integrata Verona	Oligometastatic Gastric Adenocarcinoma	Combination Product: FOLFOX and PIPAC
Intraperitoneal Aerosolized Nanoliposomal Irinotecan (Nal-IRI) in Peritoneal Carcinomatosis from Gastrointestinal Cancer	University Hospital, Ghent	Peritoneal CarcinomatosisPeritoneal Metastases Colorectal Cancer	Drug: PIPAC with Nal-IRI
PIPAC for the Treatment of Peritoneal Carcinomatosis in Patients With Ovarian, Uterine, Appendiceal, Colorectal, or Gastric Cancer	City of Hope Medical Center	Clinical Stage IV Gastric Cancer AJCC v8	Procedure: Biopsy Drug: Cisplatin Drug: Doxorubicin
